# The Effects of Joint Mobilization and Myofascial Release on Muscle Thickness in Non-Specific Low Back Pain: A Randomized Clinical Trial

**DOI:** 10.3390/jcm14082830

**Published:** 2025-04-19

**Authors:** Hafiz Muhammad Waseem Javaid, Syed Shakil Ur Rehman, Muhammad Kashif, Muhammad Salman Bashir, Wajeeha Zia

**Affiliations:** 1 Riphah College of Rehabilitation Sciences, Riphah International University, Islamabad 46000, Pakistan; shakil.urrehman@riphah.edu.pk (S.S.U.R.); wajeeha.zia@riphah.edu.pk (W.Z.); 2 School of Health Sciences, University of Management and Technology, Lahore 54000, Pakistan; shs.dean@umt.edu.pk

**Keywords:** functional disability, joint mobilization, muscle thickness, myofascial release, non-specific low back pain, pain, range of motion

## Abstract

**Background**: Non-specific low back pain is a discomfort that affects individuals at any point in their lives. This study’s aim was to determine the effects of myofascial release and joint mobilization on muscle thickness via ultrasonography in individuals experiencing non-specific low back pain. **Methods**: This double-blinded randomized clinical trial was conducted on 84 participants in three groups: joint mobilization, myofascial release, and a combination of joint mobilization and myofascial release. Data were collected during a two-week treatment regimen (days 1, 4, 8, and 12) and at a one-month follow-up. Ultrasound evaluations were used to measure the thickness of deep lumbar muscles at rest and contraction, i.e., the transverse abdominis (rTrA and cTrA) and lumbar multifidus (rLM and cLM). Repeated-measures ANOVA was utilized to analyze the follow-ups within the groups and among the groups, with post hoc tests conducted to identify specific differences. **Results:** Significant increases in muscle thickness were observed over time in the transverse abdominis, with improvements in both rTrA (right, *p* = 0.001; left, *p* = 0.001) and cTrA (right, *p* = 0.001; left, *p* = 0.008). The lumbar multifidus also demonstrated significant changes, with increases in the rLM (right, *p* = 0.001; left, *p* = 0.047) and cLM (right, *p* = 0.004; left, *p* = 0.037). However, the main effects showed no significant differences in muscle thickness among the groups. **Conclusions:** Joint mobilization demonstrated increased effectiveness in improving muscle thickness relative to myofascial release and a combination of both treatments for individuals with non-specific low back pain.

## 1. Introduction

Non-specific low back pain (NSLBP) affects many patients through pain and discomfort between the costal margin and lower gluteal folds while also potentially extending to the lower extremities. It is one of the most prevalent musculoskeletal issues worldwide and impacts 4.2% to 19.6% of all individuals globally [1,2]. NSLBP is the primary cause of disability and has a substantial impact on the worldwide disease burden. The latest Global Burden of Disease Study shows that NSLBP is the greatest disability burden agent because it accounted for 64.9 million years lived with disability (YLDs) in 2019. This significant number reveals the sweeping impact that NSLBP has on people’s quality of life and workplace production, in addition to healthcare expenses [3].

Medical expenses and absenteeism from work, together with lasting functional disabilities from NSLBP, limit people’s ability to carry out daily activities [3]. The pathophysiological presentation of NSLBP includes increased facet joint restrictions coupled with myofascial thickening and reduced lumbar flexibility compared to the healthy population, making patients more prone to secondary dysfunction of connected structures [4]. Scientists have established that NSLBP development and persistence depend on the essential role of lumbopelvic fascia because of its dysfunctions, which include inflammation, fibrosis, adhesions, fat infiltration, and other structural abnormalities [5,6,7]. The treatment of these dysfunctions with specialized care approaches remains essential for better patient recovery and decreased global prevalence of this disabling condition.

Myofascial systems in the lumbar region that symbolize the tensegrity framework, formed by the fascia and deep muscles of the back, are essential for structural equilibrium, kinematics, and movement of the body [8]. These systems, particularly through the fascia with the transversus abdominis and multifidus, have direct connections to the facet joints, facilitating movement in the lumbopelvic region and enabling force transmission between the appendicular and axial skeletons [9,10]. The transversus abdominis (TrA) and lumbar multifidus (LM) muscles, being local stabilizers, are more prone to weakness compared to the global mobilizers, e.g., erector spinae, which tend to exhibit increased activity and force generation for gross movements [11]. Consequently, restrictions within these systems can lead to joint dysfunction by limiting movement and impairing overall biomechanical function [12].

Multiple treatment approaches can be used to treat NSLBP, including pharmacological interventions, alternative medicine, and conventional treatments [13]. Manual therapies, such as JM and MFR, have gained attention in recent years because of the side effects of pharmacological interventions [14]. JM is considered an effective manual therapy approach for NSLBP; it involves gentle oscillatory movements to the joints and elicits physiological responses, such as pain relief, improved joint flexibility, reduced pain sensitivity, and modifications in muscle contraction or activity levels [15]. It modifies internal mechanical tension between vertebras [16] and assists individuals with NSLBP in temporarily alleviating discomfort [17] and impairment when compared to alternative therapies [18]. Despite variability in outcomes, JM is one of the therapeutic approaches used for treating NSLBP [19,20].

MFR is a type of manual therapy specifically targeting the myofascial system to enhance patient fitness and alleviate pain intensity, decrease muscle activity through the mechanical activation of mechanoreceptors located within the connective tissue, ultimately increase flexibility [9,10], and facilitate sliding between layers of soft tissues [8]. From a practical perspective, MFR entails gradually stretching the patient’s muscles and fascia by hand to release the myofascial system from tension and dysfunction [21]. Studies have demonstrated that even a single, isolated MFR treatment can improve the lumbar spine range of motion, lower pain levels [22], and reduce the tightness of the erector spinae and multifidus [23].

Ultrasound imaging is a non-invasive method that enables the visualization of tissue structures by using the reflection of ultrasound waves from heterogeneous tissues, allowing for a quantitative assessment of subcutaneous structures, connective tissues, and muscles [24]. Previous studies have successfully utilized ultrasonography to analyze muscle thickness in the lumbar region, both at rest and during contraction, as an indicator of muscle function and activation among healthy individuals [24,25]. However, there remains a gap in identifying the effects of manual therapy treatment approaches, i.e., JM and MFR, on the TrA and LM muscles in patients with NSLBP [26,27].

This study aimed to investigate the effects of JM, MFR, and their combination on the thickness of the TrA and LM muscles in individuals with NSLBP. The findings could enhance clinical practice by generating quantitative data on muscle thickness, offering insights for more effective treatment strategies, validating the effectiveness of ultrasonography in assessments, and promoting manual therapies as safer alternatives to pharmacological interventions.

## 2. Materials and Methods

### 2.1. Study Design and Setting

This randomized clinical trial was conducted at the Al-Razi Healthcare and Riphah Rehabilitation Center, Lahore, from 25 April 2021, to 30 November 2022, following the ethical guidelines established by the ethical committee of Riphah International University, Islamabad, Pakistan. The trial received approval from the Institutional Review Board (Ethics No. REC/RCR & AHS/21/1104), ensuring adherence to the ethical standards set by the World Medical Association and the Helsinki Declaration of 1975. Additionally, the trial was registered on ClinicalTrials.gov (NCT04860726), https://clinicaltrials.gov/study/NCT04860726 (accessed on 12 February 2025).

### 2.2. Sample Size

The sample size was primarily determined through a pilot study involving 30 participants (10 in each group) and calculated using n4studies software version 1.4. The outcome measure used for sample size estimation was the “1-month reading thickness of contracted lumbar multifidus at the L4–L5 level on the right”. By using a two-tailed independent test, we determined the expected mean change in the JM group, with *μ*_1_ = 4.04 and variance = 0.60, and the expected mean change in the MFR group, with *μ*_2_ = 4.50, variance = 0.54, Alpha (*α*) = 0.05, *Z*(0.975) = 1.96, and 1 − Alpha (*α*) Beta (*β*) = 0.20, *Z*(0.8).

$$n=\frac{{\left(Z_{\frac{\alpha }{2}}+Z_{\beta }\right)}^{2}.\left(s{²}_{1}+s{²}_{2}\right)}{\left(\mu_{1}\;-\;\mu_{2}\right)}$$

Based on these calculations, each group was planned to consist of 25 participants, with a total of 75 participants across the three groups. An additional 20% of participants were included in the study to compensate for possible attrition, resulting in a total of 90 study participants. Longitudinal investigations encounter attrition challenges in studies that need multiple sessions because participants sometimes withdraw from the research because of personal or health conditions or logistical difficulties, making them discontinue; therefore, this study included 20% extra participants beyond the study requirements to protect the statistical power when survey participants left the study. The research concluded with 6 participants withdrawing, thus resulting in a total participant count of 84 at study completion [28]. A post hoc power analysis was performed using the observed effect sizes and the final sample size to determine if the investigation had enough statistical power. The analysis showed that this study had enough power (>80%) to reach the expected effect size for the primary outcome measure. Although the observed effect sizes for some secondary outcomes were smaller than expected, these results were statistically significant. Still, they were not to be taken too seriously in terms of their clinical significance.

### 2.3. Selection Criteria

The following selection criteria were considered in this study: participants aged 20–50, diagnosed with NSLBP, who had not undergone any form of physical therapy or exercise regimen in the preceding three months, had NSLBP for less than three months, had refrained from the use of analgesics and non-steroidal anti-inflammatory drugs for the past 1 week [29], and those with a Roland Morris disability questionnaire disability rating ≥8 were included [30]. Individuals with problems such as tumors, constitutional symptoms, integumentary issues, cauda equina syndromes, or previously diagnosed with systemic issues, specific neuromusculoskeletal conditions, a recent history of trauma, prior surgical procedures, nerve blocks in the lumbosacral region, pregnant females or having given birth within the last six months, and the inability to lie prone or supine for at least twenty minutes were excluded to overcome confounders [31]. All participants underwent a thorough screening and assessment process in the standing, supine, prone, and sitting positions. This evaluation incorporated special tests, such as the straight leg raise (SLR), cross SLR, flexion, abduction, and external rotation (FABER) test, femoral nerve stretch test, and a back inspection for potential deformities. To uphold the study’s integrity, individuals with positive results on any of these assessments were excluded [29].

### 2.4. Data Collection Procedure

**Figure 1:**
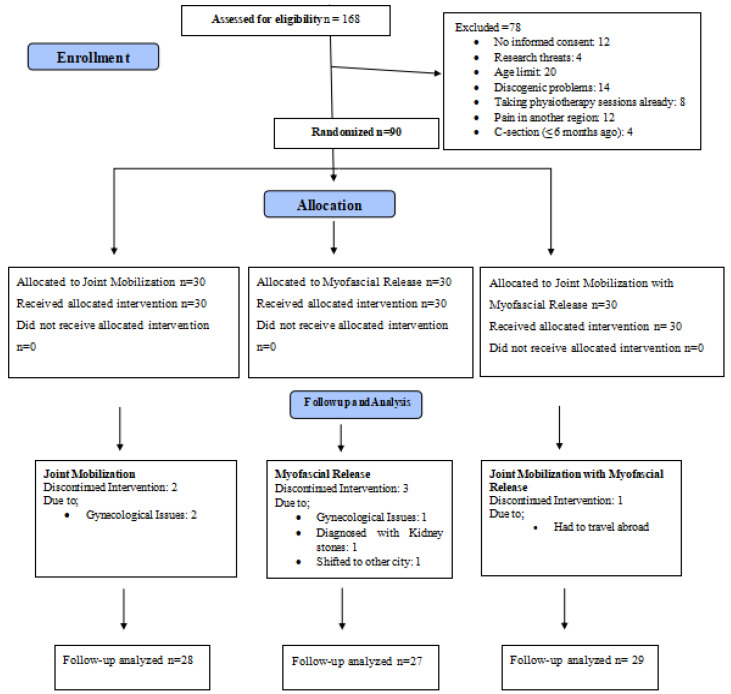
CONSORT.

Data were collected on day 1 (before and after treatment); on days 4, 8, and 12 after the intervention; and at follow-up after a month. Participants were recruited after informed consent, either through responses to flyers or referrals from general practitioners. This study adhered to the Consolidated Standards of Reporting Trials (CONSORT) guidelines, as illustrated in Figure 1. Steps were taken to maintain the anonymity and confidentiality of the data collected. The patients were randomly allocated into three groups using an online randomization tool (https://www.randomizer.org, accessed on 10 September 2022, which ensured that each participant was assigned a number. The computer-generated allocation sequence was created prior to the start of the study, following the criteria established by GD Ruxton [32]. To maintain double-blinding, both participants and assessors were blinded to group assignments, minimizing bias and ensuring the accuracy of the results. An independent assessor with more than five years of experience in manual therapy was then randomly assigned to the experimental group.

### 2.5. Outcomes

Muscle thickness was assessed using ultrasonography to evaluate the TrA and LM muscles.

#### Muscle Thickness Assessment

Ultrasound imaging was performed by the same experienced technician in B-mode with a Toshiba TA700 Medical LCD Monitor (Toshiba Medical Systems Corporation, Tokyo, Japan) ultrasound machine using a 7–15 MHz linear probe and a 2–5 MHz curvilinear probe over the TrA and LM muscles both at rest and contraction. The assessment of muscle thickness using ultrasound imaging is a reliable method, with ICCs varying from 0.88 to 0.98, the SEM ranging from 0.46 to 2.55 mm, and the MDC ranging from 1.27 to 7.30 mm [33]. Participants were in the supine position for the TrA muscle thickness at rest. The transducer was placed slightly above the iliac crest along the mid-axillary line with hips and knees extended. They were instructed as follows to assess contraction: “Raise your leg off the table without bending your knee approximately 8 inches” [34]. The LM muscle thickness at rest was measured in the prone position with a pillow under the abdomen to reduce lumbar curvature at the L4–L5 zygapophyseal joint level. For contraction, the LM thickness was assessed during a contralateral arm lift task, holding a weight proportional to body mass, similar to the mentioned position with elbows flexed at 90° and shoulders abducted at 120°. They were instructed to lift their arm approximately 2 inches off the table [31]. All procedures at rest and contraction were rehearsed once before image acquisition.

### 2.6. Intervention

A total of four treatment sessions according to the designated treatment groups, each lasting for 20 min, over a period of two weeks (days 1, 4, 8, and 12), were provided to the participants. In addition to their assigned interventions, all participants received standardized physical therapy with a heating pad for 15 min and trunk stabilization exercises, e.g., glute bridge and dead bug (each with 2 sets of 10 repetitions with 30s intervals) [5].

#### 2.6.1. Joint Mobilization

**Figure 2:**
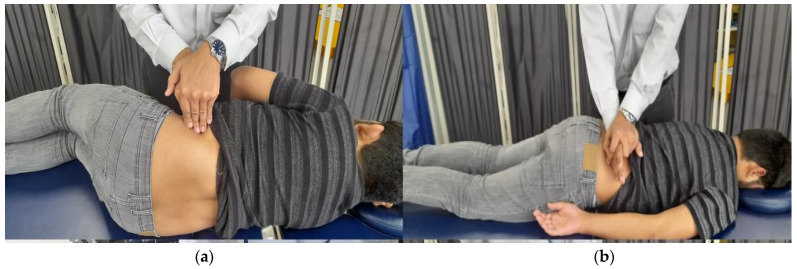
(**a**) Therapist applying lumbar joint mobilization (Maitland PA Grades III and IV). (**b**) Myofascial release to target the lateral raphe.

The participants were positioned prone, with arms either alongside the body or extending off the couch, and the head was turned comfortably to one side. The therapist applied the posteroanterior [5] glides at the L4–L5 facet joints using the hypothenar and fifth metacarpal of one hand, with the other hand superimposed. The treatment included oscillatory rocking motion of the upper trunk along a vertical axis with 3 sets of 10 repetitions each using the concept of Maitland’s Vertebral Joint Mobilization. Grade III has a large amplitude, nearing tissue stretch limits, whereas Grade IV has smaller amplitudes, pushing tissue to, or even beyond, its stretching capacity [35], as shown in Figure 2a.

#### 2.6.2. Myofascial Release

Participants adopted a side-lying position for contralateral evaluation. Utilizing ultrasound, the midpoint of the lateral raphe (LR) of the thoracolumbar fascia was identified between the TrA and the posterior musculofascial junction [26]. Manual pressure was vertically applied for 90–120 s following the technique outlined in The Myofascial Release Manual [36], as shown in Figure 2b.

#### 2.6.3. Combination of Joint Mobilization and Myofascial Release

The participants were administered an intervention involving lumbar joint mobilization (Maitland PA Grades III and IV) with myofascial release, as shown in Figure 2a,b.

### 2.7. Data Analysis

All analyses were conducted using IBM SPSS Statistics Version 26 (International Business Machines Corporation, Armonk, NY, USA). Descriptive statistics were used to summarize the mean and standard deviation. The normality of the data was assessed using the Kolmogorov–Smirnov test. A post hoc power analysis evaluated the sample size of n = 84 to determine its effectiveness in detecting observed research effects. This study reached statistical significance based on its specified α = 0.05 sample size yet produced unexpected low effect sizes that might diminish the clinical importance of the results. Repeated measures of analysis of variance (ANOVA) were utilized to analyze the follow-ups within the groups and among the groups with post hoc tests. Percentage changes in the outcome measures within each group were calculated using the mean and standard deviation. 

## 3. Results

An initial screening of eligibility was conducted on a total of 84 patients. Following the application of the selection criteria and dropout, 28 patients were allocated to the joint mobilization (JM) group, 27 to the myofascial release (MFR) group, and 29 to the joint mobilization with myofascial release (JM + MFR) group. The research comprised those with chronic lower back discomfort who fit particular qualifying criteria determined by age, BMI, and clinical evaluation. Patients without recent trauma, inflammatory joint disorders, or past spinal surgery are included in Figure 1, which indicates the participant distribution among the three intervention groups.

Table 1 shows the participants’ starting demographic traits. With no statistically significant variation among the groups (*p* = 0.212), the mean age was very comparable. While other factors, like weight (*p* = 0.382) and BMI (*p* = 0.237), showed no appreciable change, the heights of patients varied greatly among the groups (*p* = 0.026). Furthermore, similarities across all groups included daily working hours (*p* = 0.855). Based on the measured baseline disability levels using the Roland–Morris disability questionnaire (RMDQ-1), the groups’ starting points of functional impairment were similar (*p* = 0.937).

**Figure 3:**
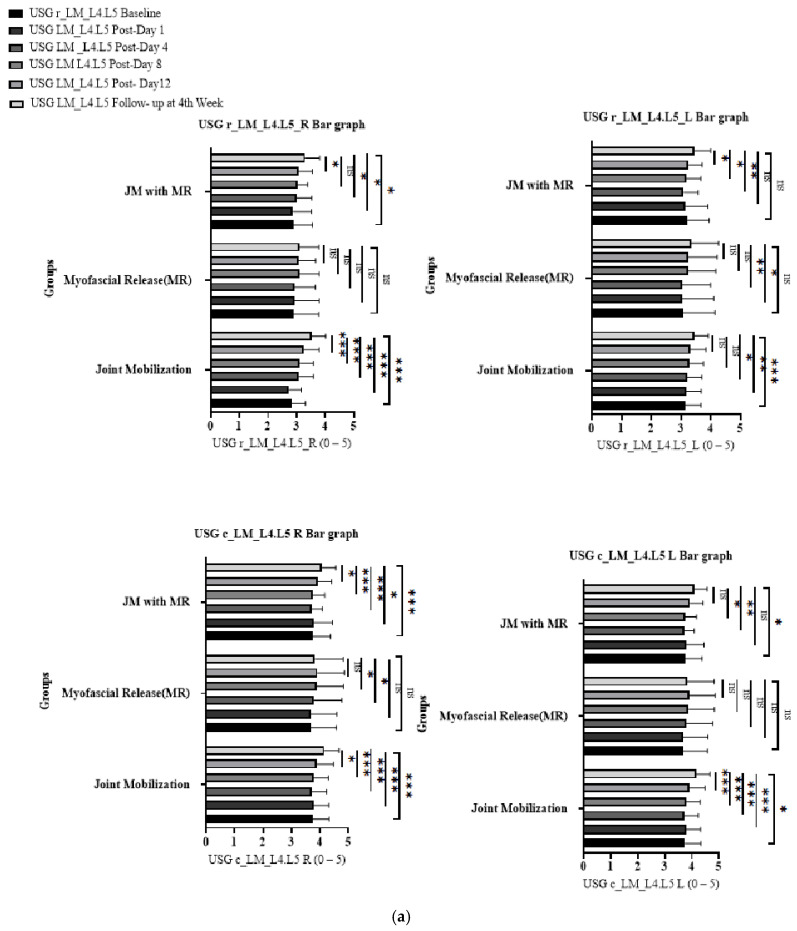

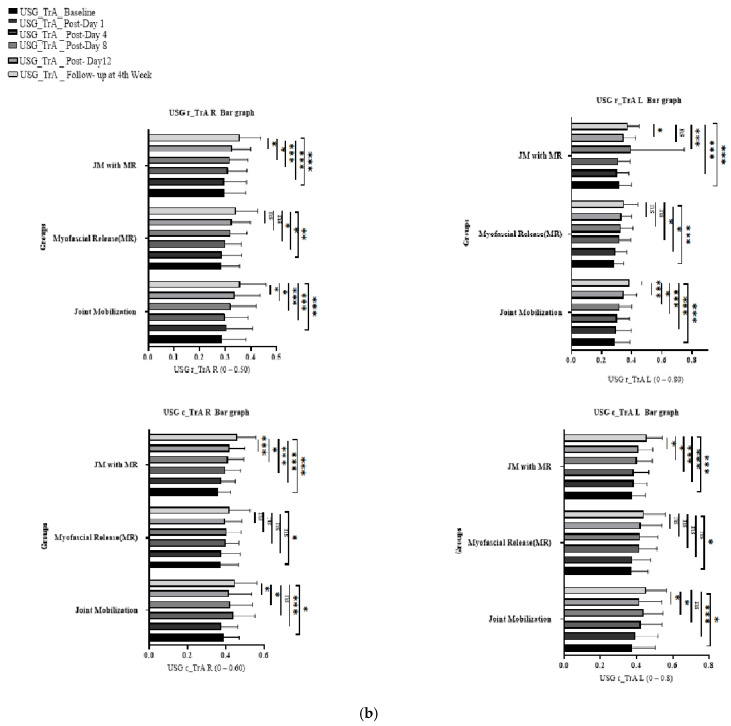
Main time effects of muscle thickness across three groups (JM, MFR, and JM + MFR) on (**a**) LM and (**b**) TrA. (0.12 (ns), 0.033 (*), 0.002 (**), <0.001 (***)).

The repeated-measures ANOVA (Table 2) results for the TrA and LM muscle measurements, both at rest and during contraction, showed statistically significant increases (*p* < 0.001) from the baseline to the follow-up assessments, particularly in the JM group. These findings indicate a positive effect of the intervention on muscle thickness across all evaluated variables, with the most notable improvements observed in the JM treatment (Figure 3).

**Figure 4:**
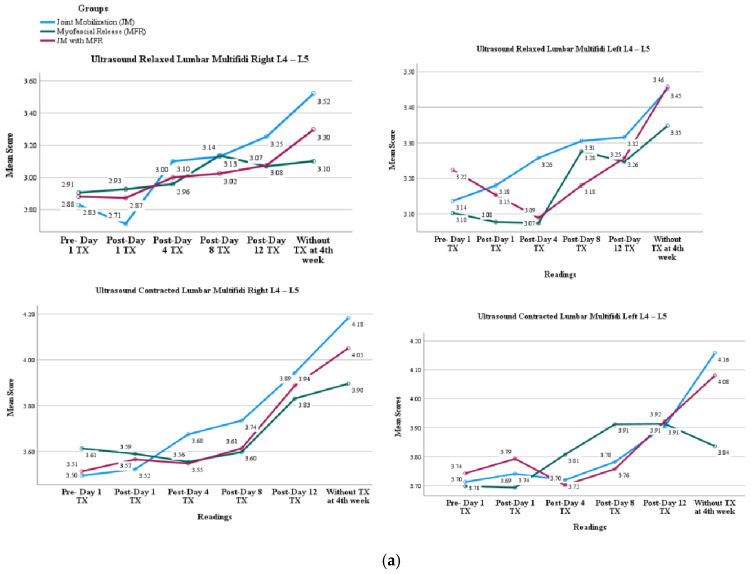

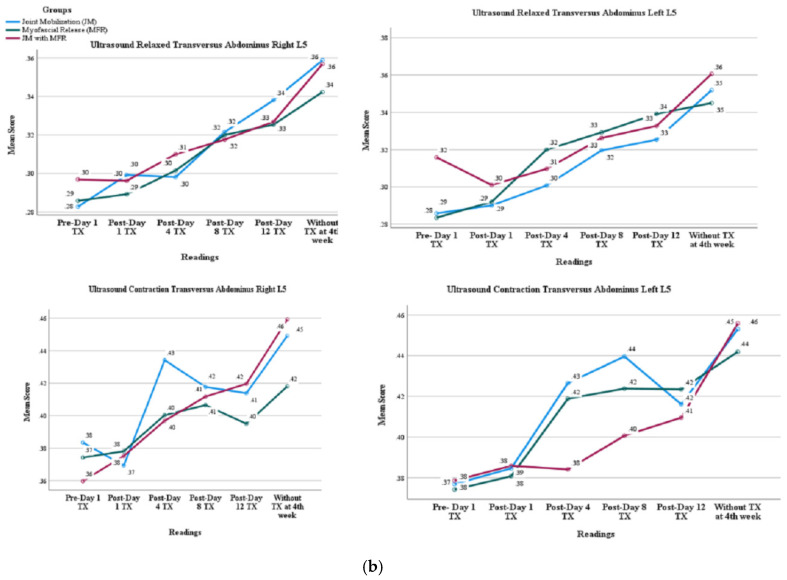
The main effects of muscle thickness across three groups (JM, MFR, and JM + MFR) on (**a**) LM and (**b**) TrA.

Table 3 displays the percentage changes in muscle activity for resting and contracting the TrA and LM across the JM, MFR, and combined JM + MFR groups. While muscle thickness improved in all groups, no significant differences were observed among the treatments, indicating similar effects across the interventions. Notably, the right TrA at L5 showed percentage changes of 21.4% for JM, 34.9% for MFR, and 33.0% for JM + MFR, highlighting the most significant improvement with MFR. Overall, these results suggest that both JM and MFR can enhance muscle thickness, but the combined approach does not significantly outperform the individual therapies (Figure 4).

## 4. Discussion

This study aimed to assess the effects of JM, MFR, and their combination (JM + MFR) on muscle thickness in individuals with NSLBP. By measuring changes in muscle thickness, this research sought to provide a clearer understanding of how these interventions may influence muscle thickness and contribute to the management of NSLBP.

Both interventions, joint mobilization and myofascial release, demonstrated positive outcomes on muscle thickness by minimizing spinal stiffness and fascial adhesions, with joint mobilization exhibiting significant effects. Joint mobilization at lumbar facet joints promotes multifidus activation directly and that of the transverses abdominis through thoracolumbar fascia by regaining spinal mobility and enhancing proprioception, neuromuscular control, and activation during functional tasks [37]. These findings are consistent with those of Choi et al., who reported significant improvements in muscle performance and lumbar stability following joint mobilization [38]. In contrast, fascial adhesions in the thoracolumbar fascia and lateral raphe, which reduce the recruitment of adjoining muscles, are decreased by MFR. Ajimsha et al. highlighted the efficacy of myofascial release in improving myofascial flexibility, blood circulation, and pain [39], which could be a contributing reason to increased muscle thickness, as seen in this study.

The joint mobilization technique combined with myofascial release operates synergistically when performed together because they use different methods of treatment. JM techniques improve joint structures, which enhance both mobility and proprioception to contribute to higher neuromuscular activation of stabilizing muscles, including multifidus and transversus abdominis [40]. The MFR technique addresses fascial restrictions, which enhances tissue elasticity while improving blood flow, making it possible to recruit muscles efficiently and reduce pain [39]. JM may generate a comprehensive treatment result when working with MFR that enhances joint mobility and neuromuscular control and creates the perfect environment for efficient muscle activation. Muscle thickness improvements in the JM + MFR group might be explained by this synergistic treatment combination, although statistical significance could not be determined between the individual interventions.

Increases in rTrA and cTrA thickness were observed in all groups: JM, MFR, and JM + MFR, yet no distinct differences among the groups were found. Statistical analysis confirmed a significant effect of time (*p* < 0.05) on muscle thickness, indicating that the interventions improved muscle thickness after a series of follow-ups. However, the main group effects and time–group interactions were not significant (*p* > 0.05), suggesting that all groups demonstrated improvements, but no single treatment consistently outperformed the others at different follow-up points. This aligns with previous research suggesting that manual interventions can enhance the activation of stabilizing muscles, though the degree of effectiveness may be similar across different treatment modalities [41]. In contrast, Lin et al. conducted a study on the thoracic spine and found a significant main group effect and time into group interactions, indicating significant differences between the mobilization and soft tissue release group [16,42]. Additionally, the current study found insignificant differences at every measurement point among the groups, which is similar to the results of Lin et al., representing a substantial temporal effect at every measured point [16].

The thickness of the rLM and cLM was significant in the JM and JM + MFR groups compared to the MFR group among the participants with NSLBP. Within-group differences indicate that the anatomical distribution of the LM in close vicinity to the lumbar facets and enhanced mobility after joint mobilization may generate increased tissue elasticity in the surrounding muscles. These results are consistent with a study focused on joint manipulation as the manual treatment approach, representing an improvement in the thickness of the LM. This may be due to g-motoneuron modulation through mobilization as one of the underlying processes of improvement [43]. Mitchell UH et al. found no association between the LM and JM among healthy patients compared to NSLBP patients. Lumbar dysfunction may be a reason for the change in the thickness of the LM that was improved after the interventions in the current study [44].

In this study, all the interventions across the TrA and LM showed percentage change improvements more dominantly on the right side, rTrA, rLM, and cLM compared to the left side and cTrA. These differences may be due to the dominant side of the body, which may be attributed to compensatory mechanisms and the connections to fascial realignments during lumbar stabilization tasks; specifically, the left TrA may engage more due to the additional load on the right side, resulting in increased muscle recruitment [45,46]. Consequently, the LM, particularly on the right side, demonstrated greater impact during day-to-day activities owing to the involvement of the right posterior back line [47]. This finding emphasizes the vital importance of focusing on the TrA and LM to improve lumbopelvic stability during complex functional movements, leading to increased muscle thickness and the activation of motor unit recruitment on the dominant side [48,49].

The transversus abdominis and lumbar multifidus [3] muscles reveal insights into physiological responses and methodological factors affecting outcomes. Notably, the right TrA (rTrA) thickness increased significantly from the baseline measurements (0.28 ± 0.09) to follow-up (0.36 ± 0.10) after joint mobilization, indicating enhanced muscle engagement linked to the intervention. However, the temporary declines observed between day 1 and day 4 could suggest short-lived fatigue or localized inflammation [50]. In the case of the right LM (rLM), an initial reduction in thickness from 3.52 ± 0.54 to 2.73 ± 0.43, followed by a recovery to 4.18 ± 0.58, corresponded with the body’s acute response to therapeutic loading and the adaptations that followed [51]. Individual variability in baseline muscle function, recovery abilities, and biomechanics may explain these patterns [52]. Moreover, the timing of assessments in relation to treatment could reflect temporary, rather than consistent, changes in muscle condition [53].

For the TrA, significant improvements in muscle thickness were observed in all treatment groups, with JM showing greater effectiveness than both JM + MFR and MFR. In contrast, for the LM, JM again outperformed JM + MFR, while no significant effects were detected in the MFR group. One reason for the effectiveness of the JM approach could be its direct impact on stabilizing the lumbar region and facilitating joint mobility through enhanced fascial attachments. This stabilization likely promotes better activation of adjacent muscles, contributing to improved outcomes [40]. In contrast, the JM + MFR group showed different results, potentially due to the combined interventions. While MFR alone did not produce significant changes in the LM, the additional benefits of JM may have amplified the effects on muscle activation and coordination. The lack of a significant change in the multifidus could be related to the small size of the LM and the limitations of the MFR technique itself. Furthermore, employing measures such as the cross-sectional area might yield more precise evaluations of muscle girth changes due to these interventions in the LM [54,55].

Statistical analysis showed that no significant differences emerged among the groups in numerous comparisons, but certain observed trends could have potential clinical importance. The participants in the JM group maintained constant advancement of TrA thickness, while the MFR participants showed reduced results in this measurement. The JM technique appeared to improve the TrA more compared to MFR despite the lack of statistical significance because it directly influences stabilizing mechanisms and fascial attachments [40]. The JM and JM + MFR intervention approaches produced larger changes in the LM thickness than the MFR approach did, demonstrating that treatments targeting the joints display superior effectiveness for deep core muscles, e.g., the TrA and LM. Clinicians might be able to use this knowledge to tailor their treatments by referring to the needs and goals of each patient in NSLBP.

The changed thickness among the TrA and LM muscles demonstrates clinical importance since these muscles maintain lumbopelvic stability during functional movements. The core stability function improves with increased TrA muscle thickness because it reduces spinal loading together with compensatory movements when performing daily activities [51]. Enhanced segmental control and proprioception arise from increased lumbar multifidus thickness, which subsequently reduces the probability of low back pain recurrences and improves functional results [43]. NSLBP patients benefit from improved outcomes that lead to diminished pain symptoms alongside better mobility and comfort in activities of daily living. The increased muscle thickness on the dominant side (right TrA and LM) signifies that these interventions could remedy the common spinal posture adaptations frequently occurring among patients with NSLBP [45,46].

Evaluating placebo effects and treatment background impacts is critical in manual therapy study design. The hands-on techniques of joint mobilization and myofascial release produce interaction effects between therapists and patients, whose natural outcome leads to psychological and physiological responses, leading to reduced symptoms. The therapeutic connection between the practitioner and patient, along with the therapist’s assurance combined with the patient’s belief in the treatment results, frequently leads to perceived therapeutic gains beyond the physical advantages of the given procedures [50]. Receiving treatment develops movement confidence while eliminating fear-avoidance behaviors and makes patients more active in their rehabilitation process. Future studies must include placebo-controlled research designs to establish the unique therapeutic effects of JM and MFR by removing the impact of placebo responses and understanding non-contact variables that enhance treatment results.

Several important findings are highlighted in this study, which makes substantial contributions to the field. It suggests a change to integrated treatment approaches in clinical practice since it shows that the combination of JM and MFR may offer better benefits for joint mobility, neuromuscular control, and muscle activation than either therapy alone. This study also focused on muscle thickness, thus offering better knowledge of physiological adaptations often neglected in favor of pain and functional results. It shows that the dominant side reacts better to therapy, suggesting that focused treatments considering this factor might improve lumbar stabilization for persons with NSLBP. Further emphasized in this study is the need to increase the TrA and LM thickness for lumbopelvic stability and pain management.

## 5. Limitations and Recommendations

This research study encountered some challenges, e.g., the non-homogeneous sample size posed a significant limitation since it undermines both internal and external validity, affecting the applicability to a wider research population. The lack of a control group hinders researchers’ ability to ascertain the appropriate order of influence among the study results. This study did not control its gender distribution, likely resulting in selection biases that hindered the identification of sex-based differences in muscle thickness alterations. Future research should ensure gender-balanced representation or examine sex-specific dynamics in smaller cohorts to identify potential patterns of muscle deformation based on gender. This study did not investigate alterations in muscle quality, including the evaluation of adipose tissue and the changes in fibrous and connective tissue inside the muscle. Future studies must investigate muscle echogenicity and conduct elastography assessments, complemented by MRI validation and conventional ultrasound thickness measures, to comprehensively understand muscle changes.

The assessment of ultrasonic measures comprised individual observations of muscle thickness; however, other metrics, such as the cross-sectional area (CSA) and pennation angle, might enhance muscle fiber analysis of the muscle architecture. The researchers ought to incorporate characteristics such as age, physical activity levels, obesity, muscle perfusion (via Doppler analysis), and involvement in resistance training to enhance study validity and reproducibility. Subsequent research should consider these factors, as they influence both muscle hypertrophy and atrophy. Future research must employ a comprehensive approach to examine participants’ routine activities in conjunction with their work settings and ergonomic practices. Research should include comprehensive physical activity records, biomechanical examinations, and lifestyle surveys to address external influences resulting from occupational postures and movement patterns.

## 6. Conclusions

JM demonstrated increased effectiveness in improving muscle thickness relative to myofascial release and a combination of both treatments for individuals with non-specific low back pain. Overall, these findings provide valuable insights into the management of NSLBP by targeting deep lumbar region muscles, specifically the transverse abdominis and lumbar multifidus.

## Figures and Tables

**Table 1:** Between-group comparison of baseline characteristics.

	Joint Mobilization (JM) (n = 28)	Myofascial Release (MFR) (n = 27)	Joint Mobilization with Myofascial Release (JM + MFR) (n = 29)	*p*-Value
Age of patient (years)	29.07 ± 6.82	32.52 ± 10.04	28.90 ± 8.47	0.212
Height of patient (cm)	167.81 ± 8.12	162.10 ± 6.56	165.12 ± 8.07	0.026
Weight of patient (kg)	70.05 ± 14.68	68.60 ± 13.23	65.08 ± 13.53	0.382
BMI of patient (kg/m^2^)	25.09 ± 4.78	26.08 ± 4.53	23.90 ± 4.97	0.237
Working hour	9.14 ± 2.90	9.33 ± 3.56	9.69 ± 4.54	0.855
RMDQ ^1^	16.55 ± 4.42	15.67 ± 4.63	16.31 ± 5.06	0.937

^1^ Body mass index: BMI; Roland–Morris disability questionnaire: RMDQ.

**Table 2:** Mean score comparison within and among groups, as well as time and group interaction.

				Assessment							RM-ANOVA	Time Main Effect		Group Main Effect		Time × Group Interaction Effect	
				Groups	Baseline	Post-Day 1	Post-Day 4	Post-Day 8	Post-Day 12	Follow-Up in 4th Week	Sig.	F-Value (Effect Size)	Sig.	F-Value (Effect Size)	Sig.	F-Value (Effect Size)	Sig.
USG ^1^	rTrA	R	L5	JM	0.28 ± 0.09	0.31 ± −0.10	0.30 ± 0.09	0.32 ± 0.10	0.34 ± 0.10	0.36 ± 0.10	0.001	19.57 (0.207)	0.000	0.244 (0.006)	0.784	0.36 (0.009)	0.904
				MFR	0.28 ± 0.07	0.29 ± 0.08	0.30 ± 0.06	0.32 ± 0.06	0.33 ± 0.07	0.34 ± 0.08	0.001						
				JM + MFR	0.29 ± 0.08	0.30 ± 0.09	0.31 ± 0.08	0.32 ± 0.70	0.33 ± 0.07	0.35 ± 0.08	0.001						
				Sig.	0.84	0.71	0.82	0.97	0.82	0.76							
		L		JM	0.28 ±0.09	0.30 ± 0.10	0.30 ± 0.08	0.32 ± 0.08	0.33 ± 0.08	0.35 ±0.07	0.001	43.76 (0.369)	0.016	0.273 (0.034)	0.273	0.39 (0.10)	0.646
				MFR	0.28 ±0.06	0.29 ± 0.08	0.32 ± 0.08	0.33 ± 0.08	0.34 ± 0.35	0.34 ±0.09	0.001						
				JM + MFR	0.32 ±0.08	0.30 ± 0.08	0.31 ± 0.08	0.40 ± 0.35	0.33 ± 0.08	0.36 ±0.07	0.227						
				Sig.	0.30	0.92	0.72	0.37	0.82	0.75							
	cTrA	R		JM	0.38 ± 0.08	0.38 ± 0.09	0.44 ± 0.11	0.42 ± 0.12	0.41 ± 0.12	0.44 ± 0.11	0.001	17.19 (0.186)	0.000	0.229 (0.006)	0.796	1.33 (0.034)	0.235
				MFR	0.37 ± 0.09	0.38 ± 0.10	0.40 ± 0.70	0.41 ± 0.07	0.40 ± 0.10	0.41 ± 0.10	0.090						
				JM + MFR	0.35 ± 0.06	0.38 ± 0.07	0.40 ± 0.08	0.41 ± 0.08	0.42 ± 0.08	0.45 ± 0.09	0.001						
				Sig.	0.44	0.99	0.15	0.78	0.62	0.33							
		L		JM	0.37 ±0.12	0.39 ± 0.13	0.42 ± 0.12	0.44 ± 0.11	0.42 ± 0.12	0.45 ±0.11	0.008	17.69 (0.191)	0.221	0.056 (0.001)	0.936	1.12 (0.029)	0.350
				MFR	0.37 ± 0.09	0.38 ± 0.10	0.42 ± 0.10	0.42 ± 0.10	0.42 ± 0.12	0.44 ±0.12	0.004						
				JM + MFR	0.37 ±0.06	0.39 ± 0.07	0.38 ± 0.08	0.40 ± 0.09	0.41 ± 0.08	0.45 ±0.09	0.001						
				Sig.	0.96	0.88	0.30	0.31	0.89	0.87							
	rLM	R	L4.L5	JM	3.52 ± 0.54	2.73 ± 0.43	3.08 ± 0.50	3.12 ± 0.47	3.25 ± 0.53	4.18 ± 0.58	0.001	1.52 (0.020)	0.001	1.490 (0.038)	0.232	1.49 (0.038)	0.231
				MFR	3.59 ± 0.84	2.92 ± 0.88	2.92 ± 0.73	3.10 ± 0.69	3.07 ± 0.59	3.89 ± 0.76	0.265						
				JM + MFR	3.51 ± 0.55	2.87 ± 0.64	3.00 ± 0.52	3.02 ± 0.35	3.08 ± 0.47	4.05 ± 0.53	0.008						
				Sig.	0.92	0.56	0.60	0.40	0.36	0.03							
		L		JM	3.14 ±0.53	3.18 ± 0.49	3.21 ± 0.49	3.28 ± 0.47	3.32 ± 0.52	3.45 ±0.47	0.047	6.34 (0.078)	0.001	0.288 (0.008)	0.751	0.846 (0.015)	0.736
				MFR	3.07 ±1.06	3.05 ± 1.05	3.05 ± 0.94	3.24 ± 0.92	3.25 ± 0.97	3.35 ±0.91	0.063						
				JM + MFR	3.22 ±0.72	3.15 ± 0.75	3.10 ± 0.49	3.18 ± 0.48	3.26 ± 0.45	3.45 ±0.53	0.093						
				Sig.	0.78	0.80	0.65	0.83	0.92	0.79							
	cLM	R	L4.L5	JM	3.53 ± 0.54	3.56 ± 0.49	3.68 ± 0.48	3.74 ± 0.51	3.95 ± 0.66	4.18 ± 0.59	0.001	17.98 (0.193)	0.000	0.316 (0.008)	0.730	1.01 (0.026)	0.417
				MFR	3.59 ± 0.84	3.58 ± 0.85	3.52 ± 0.81	3.55 ± 0.81	3.83 ± 0.70	3.89 ± 0.76	0.063						
				JM + MFR	3.51 ± 0.55	3.57 ± 0.58	3.55 ± 0.43	3.61 ± 0.44	3.89 ± 0.40	4.05 ± 0.53	0.001						
				Sig.	0.87	0.99	0.58	0.49	0.79	0.26							
		L		JM	3.74 ±0.58	3.78 ± 0.54	3.71 ± 0.53	3.78 ± 0.52	3.91 ± 0.58	4.16 ±0.51	0.003	6.19 (0.76)	0.001	0.239 (0.003)	0.890	1.102 (0.029)	0.360
				MFR	3.68 ±0.89	3.68 ± 0.91	3.80 ± 0.97	3.88 ± 0.95	3.91 ± 0.95	3.82 ±0.98	0.287						
				JM + MFR	3.74 ±0.63	3.79 ± 0.65	3.70 ± 0.38	3.76 ± 0.42	3.92 ± 0.49	4.10 ±0.48	0.037						
				Sig.	0.94	0.82	0.82	0.75	0.99	0.22							

^1^ JM = joint mobilization, MFR = myofascial release, JM + MFR = joint mobilization with myofascial release, LM = lumbar multifidus, TrA = transverses abdominis, USG = ultrasonography, rTrA = resting transverses abdominis, cTrA = contracting transverses abdominis, rLM = resting lumbar multifidus, cLM = contracting lumbar multifidus, R= right (R), L = left (L).

**Table 3:** Percentage changes and multiple comparisons.

				Percentage Change			Multiple Comparisons Test—Mean Difference with Significance		
				Joint Mobilization (JM)	Myofascial Release (MFR)	Joint Mobilization with Myofascial Release (JM + MFR)	(JM) vs. (MFR)	(JM) vs. (JM + MFR)	(MFR) vs. (JM + MFR)
				Mean ± S.D.			M.D (Sig.)		
USG ^1^	rTrA	R	L5	21.4 ± 28.3	34.9 ± 29.8	33.0 ± 35.5	0.006 (1.00)	−0.0010 (1.00)	−0.0067 (1.00)
		L		32.49 ± 51.28	30.27 ± 34.55	25.19 ± 33.59	−0.0059 (1.00)	−0.0236 (0.62)	−0.0177 (1.00)
	cTrA	R	L5	18.6 ± 24.2	25.4 ± 29.7	22.9 ± 31.2	0.016 (1.00)	0.0075 (1.00)	−0.0084 (1.00)
		L		29.56 ± 37.45	29.03 ± 32.43	22.72 ± 26.95	0.0056 (1.00)	0.0137 (1.00)	0.0081 (1.00)
	rLM	R	L4.L5	22.9 ± 26.1	18.9 ± 33.0	21.9 ± 28.5	0.075 (1.00)	−0.066 (0.87)	−0.0087 (0.99)
		L		12.36 ± 17.56	13.47 ± 29.08	13.40 ± 30.76	0.087 (1.00)	0.0476 (1.00)	−0.039 (1.00)
	cLM	R	L4.L5	15.6 ± 20.6	20.9 ± 31.0	18.2 ± 22.9	0.078 (1.00)	0.063 (1.00)	−0.016 (1.00)
		L		13.56 ± 22.04	7.65 ± 20.99	1.85 ± 24.51	0.026 (1.00)	0.004 (1.00)	−0.023 (1.00)

^1^ JM = joint mobilization, MFR = myofascial release, JM + MFR = joint mobilization with myofascial release, LM = lumbar multifidus, TrA = transverses abdominis, USG = ultrasonography, rTrA = resting transverses abdominis, cTrA = contracting transverses abdominis, rLM = resting lumbar multifidus, cLM = contracting lumbar multifidus, R = right (R), L = left (L).

## Data Availability

The data supporting the findings of this study are available upon request from the corresponding author due to privacy and ethical restrictions. No new data were generated in this study. Further inquiries regarding data access can be directed to kashif.shaffi@riphah.edu.pk or wasim8001@gmail.com.

## Abbreviations

The following abbreviations are used in this manuscript:

BMI	Body mass index
cLM	Contracting lumbar multifidus
cTrA	Contracting transversus abdominis
JM	Joint mobilization
JM + MFR	Joint mobilization with myofascial release
L	Left
LM	Lumbar multifidus
MFR	Myofascial release
R	Right
rLM	Resting lumbar multifidus
RMDQ	Roland–Morris disability questionnaire
rTrA	Resting transversus abdominis
TA	Transversus abdominis
USG	Ultrasonography
